# Medical Opinion and Movement

**Published:** 1911-08-19

**Authors:** 


### Medical Opinion and Movement.

### Medical Examination of School Children.

The report of the Blackburn Education Committee gives a detailed account of the examination of the children in the elementary schools of Blackburn. Statistics are apt to be fallacious and caution requires to be used before great stress is laid upon them. Yet although they cannot often be described as convincing, they may be suggestive. Statistics are given of the heights and weights of children described as "half-timers," *i.e.* children attending school but also engaged in some work in a factory or elsewhere. These statistics are compared with those of the heights and weights of children in the schools who are not half-timers. It is to be feared that little of definite value can be ascertained from these statistics. It appears that both classes of children in Blackburn, whether boys or girls, and whether engaged in work outside school or not, are below the average *weight*, though, curiously enough, all when between the ages of twelve and thirteen are above the average *height*. After the age of thirteen both boys and girls, whether "half-timers" or not, are below the average height as well as weight with the exception of those girls who are at work but whose work lies outside the cotton factories. If any comparison can be made between the "half-timers" and non half-timers it is a little in favour of the "half-timers." It is perhaps interesting that statistics reveal so little difference. We may perhaps suppose that children who do not go to work possess parents sufficiently well off not to be anxious to send them to work for the sake of the money they bring to aid the upkeep of the home, and that consequently they are moderately well fed without any exertion on their own part. In the case, on the other hand, of many of the half-timers, possibly material benefit is derived by themselves as the result of their work. Questions such as these complicate any deductions which may be made from statistics as to height and weight of these classes of children. A list is added to the report giving the causes of death in Blackburn during the year 1910 of children between the ages of three and thirteen years. The list is of some interest, yet it reveals the unsatisfactory character of the nomenclature of diseases used in death certificates. There is a record of a single death from lobar penumonia, five from lobular pneumonia, and five from pneumonia. The last five cases of death from pneumonia we must suppose were due to lobular pneumonia. Even the single death from true lobar pneumonia (that is not confluent broncho-pneumonia affecting the whole of one or more lobes) is in some degree a rarity in childhood, though the disease itself is compound. The death attributed to chorea and the four to rheumatic fever it may with considerable safety be said were due to pericarditis. The two cases of death from disease of the mouth appear to be obscure, while the nature of the one death from disease of the stomach is also not clear. Death from perforation of a gastric ulcer, needless to say, does occur in childhood, but is rare. There also appears to be the not uncommon difficulty presented by laryngitis, croup, and diphtheria. In the absence of bacteriological evidence it is no doubt justifiable to attribute the cause of death to laryngitis or croup, but it is to be feared that universal post-mortem examination would reduce other diseases than diphtheria, as causes of death by obstruction of the larynx, to very narrow limits.

### Individuality and Cancer.

In the report of the Imperial Cancer Research Fund fresh remarks are made upon the subject of individuality and cancer. It is stated that tumours of the "transplantable" type grow as well in normal animals as they do in animals in which spontaneous cancer has arisen. So far as the "transplantable" tumours are concerned it is obvious that the soil provided by an animal affected with cancer presents no substantial differences from that of a normal animal. In the case, however, of a cancer which has spontaneously arisen, not only can it not be inoculated into a normal animal but apparently it rarely grows in an animal in which cancer has also spontaneously arisen. Portions of a cancerous tumour, on the other hand, grow readily when inoculated into the animal in which the tumour has originated. These facts are considered to put the question of "the individuality of cancer beyond all further discussion." The teachings of naked eye morbid anatomy and microscopical examination of tissues affected with cancer in the human subject seems therefore to be borne out by experimental investigation. The report adds that the genesis and the growth of cancer are distinct phenomena which should be separately investigated. The subject of the influence of irritation is briefly referred to. The forms of irritation which predispose to cancer apparently are many and varied, but a form of irritation effective in one case is ineffective in another. Thirty-five different "tumour strains" have been now growing for over three years. It is stated that the one feature all these strains have in common is the power of continuous growth, in spite of the most divergent structure and of extremes in the rate of growth varying from an almost explosive rapidity to one much inferior to that of embryonic tissue. Study of the behaviour of these various strains is considered to yield "objective evidence of the validity of the conclusion that the cancer-cell is a biological modification of the normal cell endowed with many inherent properties of the latter."

### Chorea.

In view of the fact that the administration of arsenic by the mouth is the routine treatment for chorea, it is not surprising that the new arsenical compound, dioxydiamidoarsenobenzol, or salvarsan, should be tried in severe rebellious cases of this disease. Dr. J. Von Bokay reports the successful treatment of a case by this drug in the *Deutsche Medizinische Wochenschrift.* The patient was a girl aged eight, who had had a previous attack of chorea of moderate severity. After six weeks' treatment with Fowler's solution the patient was cured. This time, however, the affection assumed a more severe form, the motor inco-ordination was generalised and was so intense that the patient had to be kept in bed with the sides well padded to prevent traumatism. Speech was inarticulate and feeding very difficult, owing to the motor disturbance. One injection of 20 centigrammes of salvarsan was made and caused the steady and complete disappearance of all the symptoms in the course of a month. Apart from a small cutaneous necrosis at the seat of the injection, no injurious results were produced. The author draws a comparison, in evidence of the beneficial effects of the injection, between this case and another under treatment in the hospital at the same time, in which the inco-ordination was much less severe. This patient was treated with Fowler's solution, but was far from being cured more than a month afterwards.

### Diazo-reaction in Urine.

Dr. Levinson draws attention in the *Medical Record* to a source of error that may occur in connection with the diazo-reaction of Ehrlich. This reaction, it will be remembered, depends upon the red coloration of the urine, produced by adding to the urine an equal quantity of a mixture of sulphanilic acid, hydrochloric acid, and nitrate of soda, and then adding a little ammonia. Dr. Levinson points out that a similar reaction is produced when the urine contains phenolphthalein. The red coloration in this case is due simply to the addition of ammonia. In order, therefore, to exclude the possibility of the reaction being due to the presence of phenolphthalein in the urine, it is only necessary to test the urine first by adding a little alkali. If no red coloration is produced, one may conclude that the red coloration produced by the addition of the sulphanilic mixture is a true diazo-reaction. In the case of phenolphthalein being present, the diazo-reaction will still be a true one if the addition of a little acid does not make the red colour disappear. If, on the other hand, the addition of acid discolours the urine, it may be concluded that the red colour is due entirely to the phenolphthalein and the diazo-reaction is negative.

### Pneumatic Rupture of the Bowel.

An extraordinary case of accidental rupture of the intestines is reported by Dr. Andrews in *Surgery, Gynæcology, and Obstetrics*, resulting from the use of a compressed-air apparatus. The patient, a young Pole, was using the apparatus to drive the dust from his clothes, and in passing it over his trousers held it in front of the anus, without, however, applying it against himself. He at once fell to the ground and was brought to the hospital in an apparently moribund condition. His face was cyanosed, the pulse imperceptible, the abdomen enormously distended, and the neck and chest emphysematous. Passage of a rectal tube caused no escape of gas. Under local anæsthesia an abdominal incision was made, resulting in a tremendous escape of gas. The patient's condition at once improved rapidly. Five hours later laparotomy was performed, as intestinal rupture was evident. The rupture was found 12 centimetres long in the sigmoid flexure. A resection was performed with latero-lateral anastomosis. The patient recovered satisfactorily. The author has been able to collect fifteen other cases of this kind in the United States, almost all unpublished. In six of these laparotomy was performed—four died and two recovered. The remaining nine were not operated upon and all succumbed. The diagnosis of the condition of course depends largely upon the history of the accident, but the globular meteoric distension of the abdomen is very typical. The rupture generally occurs in the sigmoid, and it is frequently multiple. Resection is preferable to suturing the rupture, on account of the liability to subsequent stricture, which actually occurred in one case.

### Parathyroid.

The following case, reported by Dr. W. H. Brown in the *Annals of Surgery*, is interesting in emphasising the importance of the parathyroid glands and their relation to tetany. The patient was a young woman aged twenty-four, suffering from exophthalmic goitre. Dr. Brown removed one lobe of the thyroid, and then, as the improvement in the condition was insufficient, he removed the other. There were extensive adhesions, especially on the posterior aspect, and in view of the scepticism recently expressed in regard to the physiological value of the parathyroids, no great care was taken at the operations to preserve these organs. Four days after the second operation symptoms of tetany developed, which rapidly increased in severity. Transitory improvement was obtained, first, by subcutaneous injections of an extract prepared from fresh ox parathyroids, and again later by transplanting a parathyroid from a dog under the rectus abdominalis muscle. The symptoms, however, reappeared. Subcutaneous injections of an emulsion of dried glands had no effect. Implantations of glands from dogs and then from young oxen only produced temporary improvement. Then implantations of three parathyroids from a young monkey were tried. This time the tetanic symptoms disappeared for seventeen days. Finally, the author was able to obtain a human parathyroid from a man dying of uræmia. The gland was placed in physiological solution at 32° C. and rapidly implanted into the abdominal muscles. The result was a slow but progressive improvement. Five months later the patient was quite free from the tetanic symptoms and was much improved in health. Thyroid treatment and the administration of calcium salts failed completely. Chloroform seemed to aggravate the tetanic symptoms. In any case, it was much inferior in its effects to chloral.

### Protection from X-Ray Dermatitis.

From experiments carried out by Drs. Reicher and Lenz, under Professor Kraus of Berlin, the cutaneous ischæmia produced by subcutaneous injections of adrenalin renders the skin much less sensitive to the $x$-rays, so that the dermatitis consequent on radio-therapeutic applications may in this way be avoided. The method adopted is as follows: The solution for injection is made by adding 1 c.c. of a 1 in 1,000 adrenalin solution to 3 or 4 c.c. of normal saline containing 0.5 per cent. of cocaine. A little while before the application of the $x$-rays the solution is injected by several punctures under the skin to be exposed, so as to produce a well-marked and uniform pallor. With the skin "desensibilised" in this manner, the dose of radiations can be increased in the course of fourteen to eighteen days to double of what is sufficient in ordinary circumstances to produce an erythema. The method is especially useful for cases in which the Röntgen rays are applied to subcutaneous malignant tumours.

### Antipyrin Incompatible with Calomel.

In the *Journal of the American Medical Association* is given the upshot of certain experiments to determine the mutual reactions of these two drugs. In the presence of sodium bicarbonate the combination has led to awkward symptoms, so a mixture was prepared containing one part of calomel, three of antipyrin, and six of bicarbonate. On treating this mixture with water a bluish-grey residue is left, and a soluble mercury salt is found in the solution. This residue is partly composed of metallic mercury and of unchanged calomel, but some at least of the latter is converted into a mercuric salt. If an acid be present instead of an alkali the reaction does not occur. It appears that 2 grains of calomel in such a mixture as that first mentioned yield $\frac{1}{3}$ to $\frac{1}{2}$ grain of corrosive sublimate, a dangerous quantity in fact. If the salts were administered in a capsule the element of danger would no doubt be much lessened, since the acid contents of the stomach would suffice to prevent this reaction.

### The Uses of Pituitrin.

Two papers have appeared in the *Centralblatt für Gynäkologie* on the use of this substance in obstetric practice. In one Hofbauer recommends it as an ecbolic in incomplete abortion and for insufficient or suspended labour pains. Injected subcutaneously for these conditions, it rapidly causes normal uterine contractions, not tetanic in character. Another result is a stimulation of the bladder, or of the centre for micturition; and Hofbauer therefore tried it with success for the dysuria sometimes seen during pregnancy. In the other paper Foges and Hofstätter compare its value with that of ergotin for postpartum hæmorrhage, greatly to the advantage of the former. Besides this, they have had the opportunity of noting its effects in six cases of extra-peritoneal Cæsarean section. Thus used, it sets up firm tonic contraction of the muscle, and the operation is greatly facilitated by the absence of hæmorrhage. Pituitrin, they say, is preferable to adrenalin, because it does not increase the general blood-pressure, but only the contractility of the uterus. Another point in its favour is that the site of injection does not become painful or infiltrated, as sometimes happens after ergotin.

### Acute Sulphonal Poisoning.

A case of attempted suicide by sulphonal, reported in the *West London Medical Journal* by Dr. H. S. Sington, is noteworthy on account of the large dose swallowed and of a most important point in the treatment. A lady of thirty-eight swallowed 125 grains of sulphonal in 5-grain tablets; less than two hours later Dr. Sington saw her, at the moment when a lay friend was about to administer hot brandy. This he was just in time to prevent, and the importance of his arrival, from the patient's standpoint, may be judged when it is remembered that the solubility of sulphonal is 1 in 450 in cold water, but 1 in 90 in alcohol. The treatment consisted of a pint of coffee, which the patient had to be well roused to swallow, followed by apomorphine. Energetic stimulation with strychnine and frequent drinks of hot coffee with warmth resulted in improvement after about six hours. Subsequently an erythematous rash appeared on the face, but there were no other serious after-effects. The recovery seems to have been extraordinarily rapid after such an enormous dose; but probably the tablets had not been more than partly digested and absorbed when measures were taken for removing the poison from the stomach.

### Fluid Retention and Various Salts.

Dr. M. Pfeiffer, of Wiesbaden, read a paper at the Congress of Internal Medicine, Wiesbaden, in which he studied the retention of fluid produced in the organism by the administration of various salts. The chlorides of potassium and sodium administered daily in fairly large doses effect an increase of body weight in a few days, after which the weight declines fairly rapidly. Similar effects have been noticed after the ingestion of bicarbonate of soda. The author has had eight patients in which the phenomenon occurred. Again glycero-phosphate of magnesia has had the same result in a number of patients. In one case by this drug an increase in weight of 2 kilograms in eight days was brought about. This patient was a young woman of twenty-four, convalescent from an operation for appendicitis. Some patients, however, neither gain nor lose weight, and yet again other cases lose weight despite the ingestion of salts. The explanation of these phenomena is difficult. It is certainly not as simple as M. Heubner, of Göttingen, imagines, when he thinks that aqueous retention is favoured by the sodium ion and that it is a case not of simple osmosis but of a colloidal chemical effect.

